# Supplementary material for: Association between Periodontal Disease and Cognitive Impairment in Adults
Source: Int J Environ Res Public Health. 2023 Mar 7;20(6):4707. doi: 10.3390/ijerph20064707 (PMC10049038; doi:10.3390/ijerph20064707)
Supplement: Supplementary file 1 [file ijerph-20-04707-s001.zip › ijerph-2145377-supplementary.pdf]

Supplementary file S1: Detailed search formula in various databases

Authors	Outcome				Comparability	selection			Total
	Representativeness of the exposed cohort	Selection of the non-exposed cohort	Ascertainment of exposure	Demonstration that outcome of interest was not present at start of study	Comparability of cohorts on the basis of the design or analysis	Assessment of outcome	Was follow-up long enough for outcomes to occur	Adequacy of follow up of cohorts	
Choi et al. [1]	*	*	0	*	*	0	*	*	6/9
Demmer et al. [2]	*	0	*	*	**	*	*	0	7/9
Chen et al. [3]	*	*	*	*	**	*	*	*	9/9
Stewart et al. [4]	*	*	*	*	**	*	0	0	7/9
Tzeng et al. [5]	*	*	*	*	**	*	*	*	9/9
Sparks Stein et al.[6]	0	*	*	*	**	*	*	*	8/9

PubMed search strategy:

Search	Query	No. of hits
#1	Search: ((((((((oral pathogens) OR (oral bacteria)) OR (oral disease)) OR (oral health)) OR (mouth disease)) OR (gum disease)) OR (gum bacteria)) OR (periodontitis)) OR (gingivitis)) OR (periodontal gingivalis)	722027
#2	Search: (((((((((dementia) OR (peripheral neuroinflammation)) OR (central neuroinflammation)) OR (neurodegeneration)) OR (cognitive decline)) OR (memory loss)) OR (Alzheimer's disease)) OR (Parkinson)) OR (ALS)) OR (Vascular dementia)	616268

#3	Search: ((((((((((oral pathogens) OR (oral bacteria)) OR (oral disease)) OR (oral health)) OR (mouth disease)) OR (gum disease)) OR (gum bacteria)) OR (periodontitis)) OR (gingivitis)) OR (periodontal gingivalis)) AND ((((((((((dementia) OR (peripheral neuroinflammation)) OR (central neuroinflammation)) OR (neurodegeneration)) OR (cognitive decline)) OR (memory loss)) OR (Alzheimer's disease)) OR (Parkinson)) OR (ALS)) OR (Vascular dementia))	11768
#4	Search: ((((((((((oral pathogens) OR (oral bacteria)) OR (oral disease)) OR (oral health)) OR (mouth disease)) OR (gum disease)) OR (gum bacteria)) OR (periodontitis)) OR (gingivitis)) OR (periodontal gingivalis)) AND ((((((((((dementia) OR (peripheral neuroinflammation)) OR (central neuroinflammation)) OR (neurodegeneration)) OR (cognitive decline)) OR (memory loss)) OR (Alzheimer's disease)) OR (Parkinson)) OR (ALS)) OR (Vascular dementia)) Filters: Observational Study, Humans, English, Adult: 19+ years	112

Web of Science search strategy:

Search History:

Set	Results	Save History / Create Alert · Open Saved History	Edit Sets	Combine Sets ○ AND ○ OR · Combine	Delete Sets · Select All · ✖ Delete
# 24	5,724	#23 AND #11<br>*Indexes=SCI-EXPANDED, SSCI, A&HCI, ESCI Timespan=All years*	Edit	☐	☐
# 23	337,581	#22 OR #21 OR #20 OR #19 OR #18 OR #17 OR #16 OR #15 OR #14 OR #13 OR #12<br>*Indexes=SCI-EXPANDED, SSCI, A&HCI, ESCI Timespan=All years*	Edit	☐	☐
# 22	35,808	(TS=(Amyotrophic Lateral Sclerosis OR ALS)) *AND* **LANGUAGE:** (English) *AND* **DOCUMENT TYPES:** (Article)<br>*Indexes=SCI-EXPANDED, SSCI, A&HCI, ESCI Timespan=All years*	Edit	☐	☐
# 21	67,351	(TS=(Parkinson's disease)) *AND* **LANGUAGE:** (English) *AND* **DOCUMENT TYPES:** (Article)<br>*Indexes=SCI-EXPANDED, SSCI, A&HCI, ESCI Timespan=All years*	Edit	☐	☐
# 20	12,593	(TS=(vascular dementia)) *AND* **LANGUAGE:** (English) *AND* **DOCUMENT TYPES:** (Article)<br>*Indexes=SCI-EXPANDED, SSCI, A&HCI, ESCI Timespan=All years*	Edit	☐	☐
# 19	122,453	(TS=(dementia)) *AND* **LANGUAGE:** (English) *AND* **DOCUMENT TYPES:** (Article)<br>*Indexes=SCI-EXPANDED, SSCI, A&HCI, ESCI Timespan=All years*	Edit	☐	☐
# 18	103,995	(TS=(Alzheimer's disease)) *AND* **LANGUAGE:** (English) *AND* **DOCUMENT TYPES:** (Article)<br>*Indexes=SCI-EXPANDED, SSCI, A&HCI, ESCI Timespan=All years*	Edit	☐	☐
# 17	25,911	(TS=(memory loss)) *AND* **LANGUAGE:** (English) *AND* **DOCUMENT TYPES:** (Article)<br>*Indexes=SCI-EXPANDED, SSCI, A&HCI, ESCI Timespan=All years*	Edit	☐	☐
# 16	43,318	(TS=(cognitive decline)) *AND* **LANGUAGE:** (English) *AND* **DOCUMENT TYPES:** (Article)<br>*Indexes=SCI-EXPANDED, SSCI, A&HCI, ESCI Timespan=All years*	Edit	☐	☐
# 15	43,049	(TS=(neurodegeneration)) *AND* **LANGUAGE:** (English) *AND* **DOCUMENT TYPES:** (Article)<br>*Indexes=SCI-EXPANDED, SSCI, A&HCI, ESCI Timespan=All years*	Edit	☐	☐
# 14	4,860	(TS=(central neuroinflammation)) *AND* **LANGUAGE:** (English) *AND* **DOCUMENT TYPES:** (Article)<br>*Indexes=SCI-EXPANDED, SSCI, A&HCI, ESCI Timespan=All years*	Edit	☐	☐
# 13	2,413	(TS=(peripheral neuroinflammation)) *AND* **LANGUAGE:** (English) *AND* **DOCUMENT TYPES:** (Article)<br>*Indexes=SCI-EXPANDED, SSCI, A&HCI, ESCI Timespan=All years*	Edit	☐	☐
# 12	122,453	(TS=(dementia)) *AND* **LANGUAGE:** (English) *AND* **DOCUMENT TYPES:** (Article)<br>*Indexes=SCI-EXPANDED, SSCI, A&HCI, ESCI Timespan=All years*	Edit	☐	☐
# 11	213,996	#10 OR #9 OR #8 OR #7 OR #6 OR #5 OR #4 OR #3 OR #2 OR #1<br>*Indexes=SCI-EXPANDED, SSCI, A&HCI, ESCI Timespan=All years*	Edit	☐	☐
# 10	6,411	(TS=(periodontal gingivalis)) *AND* **LANGUAGE:** (English) *AND* **DOCUMENT TYPES:** (Article)<br>*Indexes=SCI-EXPANDED, SSCI, A&HCI, ESCI Timespan=All years*	Edit	☐	☐
[illegible]	[illegible]	[illegible]	[illegible]	☐	☐

CINAHL search strategy:

EBSCOhost

Wednesday, September 22, 2021 6:48:40 PM

#	Query	Limiters/Expanders	Last Run Via	Results
S5	(dementia OR peripheral neuroinflammation OR central neuroinflammation OR neurodegeneration OR cognitive decline OR memory loss OR alzheimer's disease OR vascular dementia OR parkinson's disease OR (amyotrophic lateral sclerosis or als)) AND (S1 AND S4)	Expanders - Apply equivalent subjects<br>Search modes - Boolean/Phrase	Interface - EBSCOhost Research Databases<br>Search Screen - Advanced Search<br>Database - CINAHL Plus with Full Text	14
S4	dementia OR peripheral neuroinflammation OR central neuroinflammation OR neurodegeneration OR cognitive decline OR memory loss OR alzheimer's disease OR vascular dementia OR parkinson's disease OR (amyotrophic lateral sclerosis or als)	Limiters - Full Text; English Language; Research Article; Exclude MEDLINE records; Human; Age Groups: All Adult; Language: English<br>Expanders - Apply equivalent subjects<br>Search modes - Boolean/Phrase	Interface - EBSCOhost Research Databases<br>Search Screen - Advanced Search<br>Database - CINAHL Plus with Full Text	2,660
S3	(oral pathogen OR oral bacteria OR oral disease OR oral health OR mouth diseases OR gum disease OR periodontal disease OR periodontitis OR gingivitis OR periodontal gingivalis) AND (S1)	Expanders - Apply equivalent subjects<br>Search modes - Boolean/Phrase	Interface - EBSCOhost Research Databases<br>Search Screen - Advanced Search<br>Database - CINAHL Plus with Full Text	560
S2	oral pathogen OR oral bacteria OR oral disease OR oral health OR mouth diseases OR gum disease OR periodontal disease OR periodontitis OR gingivitis OR periodontal gingivalis	Expanders - Apply equivalent subjects<br>Search modes - Boolean/Phrase	Interface - EBSCOhost Research Databases<br>Search Screen - Advanced Search<br>Database - CINAHL Plus with Full Text	45,574
S1	oral pathogen OR oral bacteria OR oral disease OR oral health OR mouth diseases OR gum disease OR periodontal disease OR periodontitis OR gingivitis OR periodontal gingivalis	Limiters - Full Text; English Language; Research Article; Exclude MEDLINE records; Human; Age Groups: All Adult<br>Expanders - Apply equivalent subjects<br>Search modes - Boolean/Phrase	Interface - EBSCOhost Research Databases<br>Search Screen - Advanced Search<br>Database - CINAHL Plus with Full Text	560

Supplemantary file S2: Risk of bias assessment of included studies

S2A. Risk of bias in cohort studies

S2B. Risk of bias in case-control studies:

Authors	Selection				Comparability	Outcome			Total
	Is the case definition adequate?	Representativeness of the cases	Selection of Controls	Definition of Controls	Comparability of cases and controls on the basis of the design or analysis	Ascertainment of exposure	Same method of ascertainment for cases and controls	Non-Response rate	
Montoya et al. [7]	*	*	*	*	**	*	*	0	8/9
Shin et al. [8]	*	*	*	0	*	*	*	0	6/9

S2C. Risk of bias in cross-sectional stdies:

Authors	selection			comparability	outcome	total
	Representativeness of the sample	Selection of non-exposed participants	Ascertainment of exposure		Ascertainment of outcome	
Hategan et al. [9]	0	*	*	*	*	4/6
Kamer et al. [10]	*	*	*	*	*	5/6
Noble et al. [11]	*	*	*	**	0	5/6

In order to assess the quality of studies we used the Newcastle Ottawa Scale(NOS). NOS is a star rating system where that allocates a maximum of nine stars for case-control and cohort studies across three categories: participant selection (four stars), comparability (two stars) and outcome (three stars); and maximum of six stars for cross-sectional studies across the same three categories: participant selection (three stars), comparability (two stars) and outcome (one star). Case-control and cohort studies of six or more stars and cross-sectional studies of four or more stars are considered 'high-quality' studies [12].(*) symbol means 'high' quality choices

References:

1. Choi, S.; Kim, K.; Chang, J.; Kim, S.M.; Kim, S.J.; Cho, H.J.; Park, S.M. Association of Chronic Periodontitis on Alzheimer's Disease or Vascular Dementia. *Journal of the American Geriatrics Society* **2019**, *67*, 1234–1239, doi:10.1111/jgs.15828.
2. Demmer, R.T.; Norby, F.L.; Lakshminarayan, K.; Walker, K.A.; Pankow, J.S.; Folsom, A.R.; Mosley, T.; Beck, J.; Lutsey, P.L. Periodontal Disease and Incident Dementia The Atherosclerosis Risk in Communities Study (ARIC). *Neurology* **2020**, *95*, E1660–E1671, doi:10.1212/wnl.0000000000010312.
3. Chen, C.K.; Wu, Y.T.; Chang, Y.C. Association between Chronic Periodontitis and the Risk of Alzheimer's Disease: A Retrospective, Population-Based, Matched-Cohort Study. *Alzheimers Research & Therapy* **2017**, *9*, doi:10.1186/s13195-017-0282-6.
4. Stewart, R.; Weyant, R.J.; Garcia, M.E.; Harris, T.; Launer, L.J.; Satterfield, S.; Sirnonsick, E.M.; Yaffe, K.; Newman, A.B. Adverse Oral Health and Cognitive Decline: The Health, Aging and Body Composition Study. *Journal of the American Geriatrics Society* **2013**, *61*, 177–184, doi:10.1111/jgs.12094.
5. Tzeng, N.S.; Chung, C.H.; Yeh, C.B.; Huang, R.Y.; Yuh, D.Y.; Huang, S.Y.; Lu, R.B.; Chang, H.A.; Kao, Y.C.; Chiang, W.S.; et al. Are Chronic Periodontitis and Gingivitis Associated with Dementia? A Nationwide, Retrospective, Matched-Cohort Study in Taiwan. *Neuroepidemiology* **2016**, *47*, 82–93, doi:10.1159/000449166.
6. Sparks Stein, P.; Steffen, M.J.; Smith, C.; Jicha, G.; Ebersole, J.L.; Abner, E.; Dawson, D. 3rd Serum Antibodies to Periodontal Pathogens Are a Risk Factor for Alzheimer's Disease. *Alzheimer's & dementia : the journal of the Alzheimer's Association* **2012**, *8*, 196–203, doi:10.1016/j.jalz.2011.04.006.
7. Montoya, J.A.G.; Barrios, R.; Sanchez-Lara, I.; Ramos, P.; Carnero, C.; Fornieles, F.; Montes, J.; Santana, S.; Luna, J.D.; Gonzalez-Moles, M.A. "Systemic Inflammatory Impact of Periodontitis on Cognitive Impairment." *Gerodontology* **2020**, *37*, 11–18, doi:10.1111/ger.12431.
8. Shin, H.S.; Shin, M.S.; Ahn, Y.B.; Choi, B.Y.; Nam, J.H.; Kim, H.D. Periodontitis Is Associated with Cognitive Impairment in Elderly Koreans: Results from the Yangpyeong Cohort Study. *Journal of the American Geriatrics Society* **2016**, *64*, 162–167, doi:10.1111/jgs.13781.
9. Hategan, S.I.; Kamer, S.A.; Craig, R.G.; Sinescu, C.; de Leon, M.J.; Jianu, D.C.; Marian, C.; Bora, B.I.; Dan, T.F.; Birdac, C.D.; et al. Cognitive Dysfunction in Young Subjects with Periodontal Disease. *Neurological Sciences*, doi:10.1007/s10072-021-05115-3.
10. Kamer, A.R.; Morse, D.E.; Holm-Pedersen, P.; Mortensen, E.L.; Avlund, K. Periodontal Inflammation in Relation to Cognitive Function in an Older Adult Danish Population. *Journal of Alzheimers Disease* **2012**, *28*, 613–624, doi:10.3233/jad-2011-102004.
11. Noble, J.M.; Borrell, L.N.; Papapanou, P.N.; Elkind, M.S.V.; Scarmeas, N.; Wright, C.B. Periodontitis Is Associated with Cognitive Impairment among Older Adults: Analysis of NHANES-III. *Journal of Neurology Neurosurgery and Psychiatry* **2009**, *80*, 1206–1211, doi:10.1136/jnnp.2009.174029.
12. GA Wells, B Shea, D O'Connell, J Peterson, V Welch, M Losos, P Tugwell The Newcastle-Ottawa Scale (NOS) for Assessing the Quality of Nonrandomised Studies in Meta-Analyses.
